# The influence of athletes’ gratitude on burnout: the sequential mediating roles of the coach–athlete relationship and hope

**DOI:** 10.3389/fpsyg.2024.1358799

**Published:** 2024-04-24

**Authors:** Liangshan Dong, Shisi Zou, Rong Fan, Bin Wang, Lv Ye

**Affiliations:** ^1^School of Physical Education, China University of Geoscience, Wuhan, China; ^2^School of Physical Education, Central China Normal University, Wuhan, China; ^3^School of Physical Education, Yangzhou University, Yangzhou, China

**Keywords:** athlete burnout, gratitude, coach–athlete relationship, hope, positive psychology

## Abstract

**Background:**

Athlete burnout is a widespread psychological syndrome in competitive sports, negatively impacts athletes’ competitive state and hampers the healthy development of sports organizations. With the rise of positive psychology, exploring the mechanisms of athlete psychological fatigue through the lens of psychological capital has become a focal point of recent research. This study introduces gratitude, a key element of psychological capital in positive psychology, to examine its effect on athlete burnout and its mechanism of action, with a particular focus on the sequential mediating roles of the coach-athlete relationship (CAR) and hope.

**Method:**

A cross-sectional study design was utilized, involving 483 active Chinese athletes from national training teams and professional sports teams. The sample comprised both male (n=251) and female (n=232) athletes, with an average age of 19.24 ± 3.99 years. Participants were asked to complete self-administered questionnaires, including the Gratitude Questionnaire, CAR Questionnaire, Hope Questionnaire, and Athlete Burnout Questionnaire. Structural equation modeling in AMOS 24.0 and descriptive statistics and correlation analyses in SPSS 20.0 were employed for data analysis.

**Results:**

The study revealed significant associations between athlete gratitude, CAR, hope, and athlete burnout. Notably, gratitude was found to both directly and indirectly (via CAR and hope) influence burnout levels among athletes, suggesting a sequential mediation effect.

**Conclusion:**

The findings highlight the importance of positive psychological constructs in buffering against athlete burnout. Specifically, gratitude, alongside a supportive CAR and elevated levels of hope, may play crucial roles in mitigating burnout symptoms. These insights offer promising directions for the development of targeted intervention strategies aimed at fostering athlete well-being and performance, advocating for the integration of positive psychology principles in the management and prevention of athlete burnout.

## 1 Introduction

In the dynamic environment of competitive sports, athletes are subject to an array of pressures, including stringent performance expectations, rigorous selection processes, and elevated risks of injury. These challenges contribute significantly to the prevalence of psychological burnout among athletes, a state characterized by emotional exhaustion, a diminished sense of achievement, and devaluation of sports participation (Raedeke and Smith, 2001; Gustafsson et al., 2017). Furthermore, psychological burnout adversely impacts an athlete’s well-being and performance, as well as team dynamics and cohesion (Isoard-Gautheur et al., 2016).

The advent of positive psychology has shifted focus toward the exploration of how positive mental states and emotions, notably gratitude, can serve as a buffer against athlete burnout. Gratitude, identified as a fundamental virtue within positive psychology, is posited to promote well-being and mitigate symptoms of burnout (Wood et al., 2009). Despite the broad positive outcomes associated with gratitude, such as enhanced positive emotions, well-being, prosocial behavior, and spirituality/religiosity (McCullough et al., 2002), the specific mechanisms through which gratitude impacts burnout among professional athletes remain underexplored, indicating a critical gap in research.

This study aims to address this gap by examining the influence of athletes’ gratitude on burnout within the professional sports context, with a particular emphasis on the sequential mediating roles of the coach-athlete relationship (CAR) and hope. This approach not only seeks to extend the theoretical framework pertaining to gratitude in sports psychology but also endeavors to reveal practical interventions for alleviating athlete burnout. Hence, the significance of this research lies in its potential to deepen our understanding of the role of positive psychology in sports, especially through the lens of gratitude and its mediating effects on preventing and managing athlete burnout.

In summary, this investigation into the effects of gratitude on athlete burnout among professional athletes fills an essential gap in the literature, offering a nuanced understanding of how gratitude, in conjunction with supportive relationships and hope, can form a vital component in the psychological toolkit against burnout, ultimately fostering athletes’ well-being and success in competitive sports.

## 2 Theoretical background and research hypotheses

Within the competitive sports domain, athlete burnout is characterized as a decrement in psychological functioning, precipitated by the continuous depletion of mental and physiological resources due to internal and external pressures, absent sufficient recovery. Manifestations of this condition include three primary dimensions: emotional/physical exhaustion, reduced sense of personal accomplishment, and a devaluation of sports participation (Zhang et al., 2006). Table 1 presents the archetypal symptoms associated with each dimension. Previous research has demonstrated that burnout can have detrimental effects on athletes’ physical and mental health, potentially impairing performance, undermining social relationships, and leading to a discontinuation of sports participation (Raedeke and Smith, 2001; Zhang, 2010; Zhang et al., 2014).

**Table 1:** A synopsis of typical symptoms of burnout.

Dimension	Typical symptoms
Emotional/Physical Exhaustion	“I’ve lost most of my enthusiasm. I just go through the motions during training to satisfy my own expectations.” “My body feels stiff all over, with aching waist and back.” “I feel completely drained, as if nothing matters anymore.”
Reduced sense of accomplishment	“There was a time when I simply did not want to train anymore, perhaps because the plan was inappropriate. I felt I could not improve, and mentally I felt defeated.” “As I get older and reach my potential limits, I start to think that maybe this is the best I can achieve.” “Day after day, year after year, I have no idea when it will all end.” “Sometimes my coach puts me down, and I begin to doubt whether I am cut out for swimming.”
Negative sport evaluation	“Sometimes, not winning a championship seems irrelevant; I just feel that finishing is enough.” “Swimming is exhausting and monotonous, while playing badminton or basketball is much more enjoyable.” “My body is constantly injured, which affects my training and prevents me from improving my performance. I’m at a loss; I feel I can neither quit nor continue, especially with so many people watching.”

The above symptoms were identified through interviews with elite swimmers.

Research into athlete burnout has historically employed Smith’s (1986), viewing burnout as a culmination of chronic stress. Moreover, investigators have deployed various theoretical frameworks to explore this phenomenon, including the Negative Training Stress Response Model, the Identity Development and External Control Model, and the Athletic Commitment Model. The advent of positive psychology has introduced a novel perspective, incorporating constructs of psychological capital to examine the underlying mechanisms of athlete burnout, indicating a critical shift in contemporary research trajectories (Zhang et al., 2014; Ye et al., 2016b).

Theoretical research suggests that gratitude may help alleviate athlete burnout. Fredrickson (2004) posited that gratitude, a positive emotion, has the potential to broaden individuals’ thought processes and foster creative thinking, as proposed by the Broaden-and-Build Theory. This cognitive broadening can lead to novel approaches in expressing gratitude, reciprocating to others, developing loving and thankful skills, and building friendships and social networks. These resources, in turn, become enduring personal assets that enhance resilience to stress and adversity. Consequently, they are less likely to resort to negative coping strategies such as avoidance, self-blame, substance abuse, or denial, which could mitigate the adverse effects of athlete burnout. Empirical research substantiates the adaptive benefits of gratitude in reducing psychopathological symptoms, such as depression and post-traumatic stress disorder, significantly improving subjective well-being and self-efficacy, and boosting physical health (Tennen et al., 2009; Wood et al., 2009). Furthermore, studies have found that gratitude improves sleep quality, which, in turn, boosts physical health (Wood et al., 2009). It is also a significant predictor of a decrease in symptoms like dizziness and headaches (Froh et al., 2009).

Considering the established research, our study seeks not merely to reaffirm the negative correlation between gratitude and athlete burnout but to delve deeper into this relationship. Specifically, we aim to explore how gratitude interacts with other psychological constructs within the athletic context and its impact on the multifaceted nature of burnout. Therefore, this paper advances the hypothesis:

*H1*: Gratitude is a significant negative predictor of athlete burnout, with our study providing further insight into its role and interactions within the context of competitive sports.

Previous studies have shown that the onset and progression of athlete burnout are associated with a spectrum of physiological, psychological, and sociological factors. The coach-athlete relationship (CAR) and hope are recognized as pivotal mediators in how gratitude might affect athlete burnout. CAR involves a dynamic interaction of emotional, cognitive, and behavioral elements between coaches and athletes. Meanwhile, hope refers to the experiential success resulting from the synergistic operation of pathway and agency thought processes during the pursuit of goals, representing cognitive and behavioral inclinations.

The moral affect theory of gratitude highlights the importance of moral motivation, suggesting that feelings of gratitude can lead to increased prosocial behaviors and motivations, such as altruism, care, sharing, and forgiveness. It also prompts a proactive search for opportunities to reciprocate to benefactors (McCullough et al., 2001). Individuals with high levels of gratitude tend to experience fewer interpersonal conflicts and societal obstacles (Gan, 2012). Algoe et al. (2008) examined the impact of gratitude among sorority members and found that greater gratitude in recipients led to better quality in establishing and maintaining interpersonal relationships. Further, Algoe et al. (2012) discovered that expressions of gratitude in one romantic partner significantly enhanced the perceived responsiveness of the other partner, which was predictive of relationship improvements over a six-month trajectory.

In the field of athletics, athletes with high levels of gratitude seek to understand their coaches’ perspectives during crises within the CAR. They appreciate the coach’s dedication and efforts and seek to mend the relationship (Wang et al., 2014). Qualitative studies on athlete burnout have identified that a harmonious CAR, characterized by effective communication, active listening, and empathetic concern from the coach, can provide athletes with greater social support, thereby mitigating the negative impact of burnout attributed to sports activity (Cresswell and Eklund, 2005). Moreover, positive social interactions, such as valuable advice, timely encouragement, and assistance, have been found to correlate negatively with sports-related athlete burnout (DeFreese and Smith, 2014). In summary, gratitude not only promotes harmonious interpersonal relationships but also fosters the development of the CAR, which is closely associated with athlete burnout. Consequently, this paper proposes the hypothesis:

*H2*: The CAR mediates the effect of athletes' gratitude on their burnout.

The broaden-and-build theory of positive emotions posits that gratitude can lead individuals to positively assess their present and future, fostering prosocial behaviors that foster social cohesion and strengthen interpersonal resources (Fredrickson and Branigan, 2005). These interpersonal resources, in turn, expand individuals’ coping strategies when faced with stress, aligning with the pathways thinking aspect of hope theory. Additionally, gratitude reinforces motivation; individuals with high levels of gratitude report greater recognition of social support from others, including parental upbringing, coaching, and friendship. This increased recognition is manifested as sustained passion and motivation during training and competitions, in harmony with the agency thinking aspect of hope theory. Individuals with high hope levels, who exhibit strong agency and pathways thinking, tend to view stressors as challenges and are more likely to engage in positive actions (Snyder, 2000). Furthermore, research indicates that hope alleviates burnout among college athletes, enhances achievement motivation, and stimulates learning interest (Xie et al., 2016). In sports, hope is inversely related to the three dimensions of burnout; athletes with higher hope levels report significantly lower burnout scores. Moreover, hope not only directly reduces sports-related burnout but also serves as an indirect influence through the mediating effects of positive emotions and perceived stress (Gustafsson, 2010; Gustafsson et al., 2011). Therefore, this paper proposes the hypothesis:

*H3*: Hope mediates the relationship between athletes' gratitude and their burnout.

Hope emerges from stable and secure attachment relationships and is closely connected to social connectedness (Snyder, 2002). As social groups develop, they commonly establish ideologies and norms. To align with the group’s collective standards, individuals engage in cooperation within the group and intra-group competition, which simultaneously enhances their agency thinking. Additionally, within social groups, members offer mutual support and actionable advice, aiding in the identification of concrete methods to achieve collective goals, thus enhancing individuals’ pathways thinking. A harmonious and effective CAR promotes more positive and effective communication (Jowett, 2012). Such a relationship significantly increases an athlete’s hope level and, through hope, increases satisfaction with athletic performance while reducing the negative effects of external pressures (Ye, 2016b). There is a clear link between athletes’ gratitude and the CAR, which affects the athletes’ hope level, and consequently, hope predicts athlete burnout. Consequently, this study proposes the following hypothesis:

*H4*: The CAR and hope act as serial mediators between athletes' gratitude and athlete burnout.

## 3 Methods

### 3.1 Research design and sampling method

This study adopted a convenience sampling strategy to select athletes from national training teams and professional sports teams across varied provinces and cities, including Beijing, Hubei, Zhejiang, Heilongjiang, Guangdong, and Yunnan. In total, 502 questionnaires were distributed, and 483 valid responses were received, resulting in an effective response rate of 96.2%. The participant demographic was composed of 251 males (52.0%) and 228 females (47.2%), with gender information missing for 4 participants. Additionally, 11 participants (2.3%) did not specify their competition level. The average age of the athletes was 19.24 years (SD = 3.99), and the average training duration was 6.86 years (SD = 3.55). Athletes’ competition levels varied, including secondary level (40 participants), first level (218 participants), national master level (180 participants), and international master level (34 participants), with 11 participants not specifying their level.

The survey included a broad range of sports disciplines, such as marathon, martial arts, gymnastics, shooting, archery, clay pigeon shooting, cycling, triathlon, modern pentathlon, swimming, canoeing, middle and long-distance running, weightlifting, basketball, volleyball, boxing, judo, taekwondo, wrestling, high jump, tennis, and equestrian. The convenience sampling method facilitated rapid and efficient access to a diverse group of professional athletes, offering a practical solution amidst constraints of resources and time, despite the potential limitations in statistical representation inherent to this sampling method. Our direct engagement with athletes at their training bases and schools enhanced the depth and authenticity of the collected data.

### 3.2 Data collection procedure

The data collection process was rigorously designed to adhere to ethical guidelines, safeguarding the privacy and confidentiality of participant data. This study received ethical clearance from the Institutional Review Board (IRB) of Central China Normal University, emphasizing our commitment to conducting research with the utmost integrity and respect for participant welfare. Data collection was executed on a team basis using a group testing method, allowing for an extensive reach across a diverse range of athletes. Prior to data collection, explicit consent was obtained from both team leaders and athletes, ensuring their informed participation. Furthermore, participants were compensated for their valuable contribution to the research.

The task of collecting data was entrusted to graduate students specializing in sports psychology, all of whom had received rigorous training to perform this role efficiently. This team’s expertise guaranteed the precision and effectiveness of the data collection process. To protect participant privacy, all questionnaires were completed anonymously. Participants were assured of the strict confidentiality of their responses, which would be used solely for scientific analysis. Detailed instructions were provided to encourage thorough and independent responses, thus ensuring the data’s authenticity and reliability. Each participant was given approximately 20 min to complete the questionnaire, which was then immediately collected on-site to maintain data integrity.

Employing an on-site data collection strategy not only improved the response rate but also the accuracy and reliability of the collected data, thereby significantly enhancing the research’s quality and integrity. Our systematic approach in recruiting participants actively engaged in structured training environments, coupled with a rigorous review of returned questionnaires, further solidified the validity of our findings. This meticulous attention to ethical standards and data collection methodology underscores our dedication to producing credible and ethically sound research outcomes.

### 3.3 Measures

#### 3.3.1 Gratitude questionnaire

The study employed the Gratitude Questionnaire (GQ) as adapted by Chen and Kee (2008), which was originally developed by McCullough et al. (2002). This scale employs a 5-point Likert scale (1 = strongly disagree to 5 = strongly agree), where higher scores reflect higher levels of gratitude. The questionnaire is composed of 5 items, including “Listing everyone I feel grateful to during my sporting career would be a lengthy process,” with the third item being reverse-scored. Confirmatory factor analysis indicated the following results: χ2/df = 12.34, indicating good structural validity for this version of the questionnaire. The original English version of the GQ had a reliability coefficient of 0.87 (McCullough et al., 2002). In the present study, the overall reliability of the Gratitude Scale was found to be 0.80, indicating good reliability. The GQ total score showed a moderate correlation with several theoretical constructs relevant to the scale (namely happiness, optimism, agreeableness, and extraversion), which suggests good criterion-related validity (Chen et al., 2009).

#### 3.3.2 Athlete burnout questionnaire

The study utilized the Athlete Burnout Questionnaire (ABQ) in its form revised by Zhang et al. (2010), which was originally developed by Raedeke and Smith (2001). The scale employs a Likert 5-point scale (1 = never; 2 = rarely; 3 = sometimes; 4 = often; 5 = always), where higher scores denote higher levels of psychological burnout, and lower scores reflect lower levels. It comprises 15 items, such as “Training tires me out so much that I do not have the energy to do other things,” and “I am unable to concentrate during competitions as I used to,” and includes three subscales: emotional/physical exhaustion, reduced sense of accomplishment, and devaluation in sport. These subscales account for 61.66% of the variance. Confirmatory factor analysis utilizing the maximum likelihood estimation on a first-order three-factor model of the Athlete Burnout Questionnaire yielded: χ^2^/df = 4.29, RMSEA = 0.08, SRMR = 0.05, GFI = 0.91, NFI = 0.87, CFI = 0.90, IFI = 0.90, suggesting good construct validity for this iteration. The subscales’ reliabilities for emotional/physical exhaustion, reduced sense of accomplishment, and sport devaluation, as translated into Chinese by Lu et al. (2006), were 0.88, 0.87, and 0.70, respectively. The scale’s overall reliability was measured at 0.78 in this study, with the subscales for emotional/physical exhaustion, reduced sense of accomplishment, and sport devaluation recording reliabilities of 0.78, 0.78, and 0.62 respectively, confirming good reliability. The scale demonstrated a significant positive relationship against the Chinese version of the ABQ translated by Lu et al. (2006), supporting good criterion-related validity.

#### 3.3.3 CAR questionnaire

The study employed the CAR Questionnaire (CART-Q) following the revisions of Zhong and Wang (2007), adapted from the Greek version of the CART-Q developed by Jowett and Ntoumanis (2004). This questionnaire utilizes a Likert 5-point scale (1 = strongly disagree; 2 = disagree; 3 = somewhat agree; 4 = mainly agree; 5 = strongly agree) for scoring, with higher scores signifying more positive coach-athlete relationships (CARs) and lower scores denoting less satisfactory relationships. It features 15 items, such as “I am loyal to my coach and am willing to maintain a long-term cooperation with him,” and “I am open to my coach’s advice and suggestions,” covering three dimensions: closeness, commitment, and complementarity, which account for 64.525% of the variance. Confirmatory factor analysis revealed: χ2/df = 4.98, RMSEA = 0.081, SRMR = 0.049, GFI = 0.90, NFI = 0.89, CFI = 0.91, IFI = 0.91. The original CAR Questionnaire demonstrated reliabilities for closeness, commitment, complementarity, and compliance of 0.87, 0.82, 0.88, and 0.93, respectively. In this study, the dimensions’ reliabilities were measured at 0.85, 0.86, 0.81, and 0.84, respectively, confirming the questionnaire’s good reliability. The overall scale score demonstrated significant correlations with two criterion items from the Greek version of the CART-Q (0.689 and 0.696), suggesting good criterion-related validity of the translated version of the questionnaire.

#### 3.3.4 Trait hope scale

The Trait Hope Scale (THS), following the revisions by Chen et al. (2009) based on the original scale by Snyder et al. (1991), was employed in the study. The scale comprises 12 items, such as “I can think of many ways to get out of a bind” and “I have been successful in my athletic career,” and is comprised of two dimensions: agency thinking and pathways thinking. It employs a Likert 5-point scale (1 = strongly disagree; 2 = disagree; 3 = somewhat agree; 4 = agree; 5 = strongly agree), with higher scores reflecting greater levels of hope. The confirmatory factor analysis yielded: χ^2^/df = 3.81, RMSEA = 0.076, SRMR = 0.042, GFI = 0.96, NFI = 0.94, CFI = 0.96, IFI = 0.96. The English version of the THS demonstrated reliabilities across agency and pathways thinking domains of 0.74 and 0.84, respectively. In the current study, the questionnaire exhibited an overall reliability of 0.82, with reliabilities for agency and pathways thinking at 0.69 and 0.78, respectively, and exhibited a test–retest correlation coefficient of 0.80, indicating consistency with the reliability of the English version of the Hope Scale. Validity testing revealed that agency and pathways thinking dimensions of the Hope Scale were significantly positively correlated with a proactive coping approach and significantly negatively correlated with a passive coping style, consistent with international research findings (Woodward et al., 2006), indicating good criterion-related validity of the scale.

### 3.4 Data analysis

Data were organized, processed, and analyzed using SPSS 20.0 and AMOS 24.0 software. Beyond descriptive statistics and bivariate correlations, the study primarily employed Structural Equation Modeling (SEM) as the method of data processing, with the significance level established at α = 0.05. While these components—emotional/physical exhaustion, reduced sense of achievement, and negative sports appraisal—are facets of burnout, their distinct contributions must be considered and not aggregated. The utilization of Zhang et al.’s (2010) weighted total score formula for burnout (Burnout Weighted Total Score = *Z*-score for Reduced Sense of Achievement × 0.47 + *Z*-score for Emotional/Physical Exhaustion × 0.21 + *Z*-score for Negative Sports Appraisal × 0.32) facilitated the derivation of the composite burnout score. These scores were additionally subjected to individual analyses for each dimension. The research not only examined the mediating roles of the CAR and hope between gratitude and the composite burnout score but also their intermediary functions between gratitude and the three discrete dimensions of burnout. These analyses elucidated the complex interplay among these variables, contributing to a deeper understanding of athlete burnout.

## 4 Results

### 4.1 Control and test for common method bias

Data were collected through self-report measures in this study, which raises the potential for common method bias. To mitigate this, the administration of the measures included imposing strict procedural controls regarding the data’s confidentiality, anonymity, and exclusive use for scientific research. Furthermore, Harman’s single-factor test was applied for analytic examination (Podsakoff et al., 2003; Zhou and Long, 2004). This method involved loading all measurement items of the study variables into a single factor to create a one-factor model and contrasting it with the fit indices of an 11-factor model that aligned with the theoretical dimensions. The results indicated that the fit indices for the 11-factor model (χ^2^ = 1959.39, df = 979, χ^2^/df = 2.00, RMSEA = 0.05, CFI = 0.90, IFI = 0.90, TLI = 0.88) were significantly better than those for the single-factor model (χ^2^ = 6208.24, df = 1,034, χ^2^/df = 6.00, RMSEA = 0.10, CFI = 0.44, IFI = 0.44, TLI = 0.41), which suggests that a serious common method bias is unlikely in the current study.

### 4.2 Correlation analysis of gratitude, CAR, hope, and burnout

As presented in Table 2, upon controlling for demographic variables (gender, age, years of athletic participation, and level of competition), gratitude, CAR, and hope showed a significant negative correlation with burnout. Moreover, gratitude and CAR demonstrated a significant positive correlation with hope, and gratitude was significantly positively correlated with the CAR. The absolute values of the correlation coefficients among the study variables ranged between 0.32 to 0.50, indicating their appropriateness for further analysis. The mean scores for gratitude and the CAR were notably high, potentially reflecting the influence of social desirability effects, while the limited variability could be attributed to a ceiling effect.

**Table 2:** Correlation coefficients among gratitude, CAR, hope, and burnout (*N* = 483).

	*M*	SD	1	2	3	4
1. Gratitude	4.12	0.75	1.00			
2. CAR	4.01	0.72	0.50***	1.00		
3. Hope	3.46	0.62	0.41***	0.33***	1.00	
4. Burnout	2.47	0.63	−0.41***	−0.39***	−0.32***	1.00

*p < 0.05, **p < 0.01, ***p < 0.001; the same below.

### 4.3 Examination of the mediating effect of CAR and hope between gratitude and burnout

Following the recommendation of Fang et al. (2012), the percentile Bootstrap method with bias correction offers superior statistical power compared to the traditional Sobel test. Consequently, this study employed the SPSS macro program PROCESS, developed by Hayes (2013),^1^ and controlled for demographic variables including gender, age, duration of sports participation, and level of athletic competition. Mediation effects were evaluated through a structural equation model, based on 5,000 bootstrap samples to establish 95% confidence intervals.

Preliminary results, as shown in Table 3, indicated that gratitude maintained a significant positive influence on CAR (*β* = 0.52, *p* < 0.001). When gratitude and CAR were predictors of hope, gratitude maintained a significant positive influence (*β* = 0.27, *p* < 0.001), and CAR also displayed a significant positive impact (*β* = 0.19, *p* < 0.001). When gratitude, CAR, and hope were introduced into the regression equation concurrently, each variable exhibited a significant negative predictive influence on burnout (*β* = −0.23, *p* < 0.001; *β* = −0.21, *p* < 0.001; *β* = −0.19, *p* < 0.001), indicating a significant mediating role of CAR and hope in the relationship between gratitude and burnout.

**Table 3:** Regression analysis overview for testing the mediation effects of CAR and hope between gratitude and burnout.

Regression equation		Overall model fit			Regression coefficient significance	
Outcome variable	Predictor variable	*R*	*R*^2^	*F*	*β*	*t*
CAR	Gratitude	0.52	0.28	28.10***	0.52	11.70***
Hope	Gratitude	0.51	0.26	28.89***	0.27	5.29***
	CAR				0.19	3.58***
Burnout	Gratitude	0.50	0.25	18.54***	−0.23	−4.33***
	CAR				−0.21	−3.42***
	Hope				−0.19	−3.56***

All variables were standardized before being entered into the regression equation.

**Figure 1:**
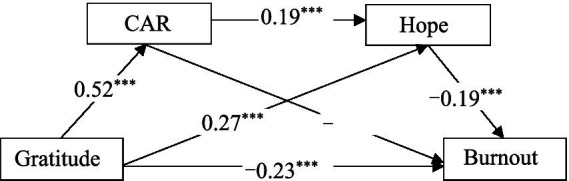
Mediation model of CAR and hope between gratitude and burnout. Solid lines indicate significant paths; dashed lines indicate non-significant paths.

Furthermore, detailed scrutiny of the mediation effects, as presented in Table 4, showed that the total indirect effects produced by CAR and hope did not include zero within the 95% Bootstrap confidence interval, indicative of a significant mediating effect by the two variables between gratitude and burnout. The mediation effect comprises three indirect effects: (1) The first, generated by the path “Gratitude → CAR → Burnout,” with the confidence interval excluding zero, indicates a significant CAR mediation between gratitude and burnout (−0.11, contributing to 26.83% of the total effect); (2) the second, generated by the path “Gratitude → Hope → Burnout,” with the confidence interval excluding zero, indicates a significant hope mediation between gratitude and burnout (−0.05, contributing to 12.20% of the total effect); and (3) the third, generated by the path “Gratitude → CAR → Hope → Burnout,” with the confidence interval excluding zero, indicates partial mediation by CAR and hope between gratitude and burnout (−0.02, contributing to 4.88% of the total effect). Based on these results, a serial mediation model as depicted in Figure 1 can be constructed, accounting for 25% of the variance in burnout.

**Table 4:** Overview of bootstrap analysis for the mediating effects of CAR and hope between gratitude and burnout.

Path	Standardized effect	Proportion of total effect	Boot standard error	95% Confidence interval		Significance
				Lower limit	Upper limit	
Total effect	−0.41	—	0.04	−0.49	−0.33	Significant
Total indirect effect	−0.18	43.90%	0.03	−0.24	−0.12	Significant
Gratitude → CAR → Burnout	−0.11	26.83%	0.03	−0.17	−0.05	Significant
Gratitude → Hope → Burnout	−0.05	12.20%	0.02	−0.09	−0.02	Significant
Gratitude → CAR → Hope →Burnout	−0.02	4.88%	0.01	−0.04	−0.01	Significant

## 5 Discussion

This study investigated the association between athlete gratitude and burnout, considering the perspectives of CARs and hope, and the mechanisms underlying this relationship. The findings indicate a significant negative correlation between athlete gratitude and burnout, with additional regression analysis demonstrating that athlete gratitude significantly predicts lower levels of burnout, thereby supporting Hypothesis H1. Athlete gratitude was also identified as being significantly positively correlated with the CAR; subsequently, a strong CAR was observed to significantly predict lower burnout, thus confirming Hypothesis H2. The mediation analysis revealed that the CAR and hope mediate the influence of athlete gratitude on burnout. This mediation effect unfolded via two pathways: the independent mediating role of hope and the sequential mediating effect of the CAR to hope, thereby validating Hypotheses H3 and H4. In summary, all four hypotheses proposed in this study have been corroborated by empirical evidence.

### 5.1 The direct effects of gratitude on burnout

Gratitude, recognized as a positive and affirming emotion, can broaden an individual’s scope of thought and action. Individuals who habitually practice gratitude tend to focus on the positive and pleasant aspects of life, enhancing their ability to recover from the negative effects of adverse events (Fredrickson, 2004). According to gratitude coping theory, those with a strong propensity for gratitude are more likely to engage in positive coping strategies when faced with challenging or risky situations (Fredrickson and Cohn, 2008). These individuals view everything they have, including life itself, as a gift, meeting life’s challenges with heightened positivity and optimism, which facilitates better social adaptation and well-being (Wood et al., 2007, 2008, 2009).

In the context of sports, gratitude can broaden athletes’ cognitive and behavioral patterns, bolster personal resources, and provide the essential material and psychological support necessary to strengthen resilience against setbacks and diminish the negative impact of distressing emotions. This enhancement of psychological well-being contributes to the prevention of burnout. Empirical evidence suggests that athletes’ gratitude leads to greater investment in their sport and adherence to ethical behavior, thereby fostering improved engagement in training and competition, superior performance, and recognition from coaches and peers (Wang et al., 2014; Ye, 2016). Additionally, athletes with a pronounced sense of gratitude are more apt to attribute their successes to the collective efforts of their support networks, including their country, family, coaches, and teammates. Confronted with competitive stress or the adversities of life, such athletes typically embrace an optimistic and proactive approach, successfully circumventing negative appraisals in sports.

### 5.2 The mediating effects of the CAR and hope on the relationship between gratitude and burnout

In the nexus of interpersonal dynamics, the Coach-Athlete Relationship (CAR) acts as a critical bridge. Gratitude fosters the development of a harmonious CAR, which is instrumental in building and sustaining social bonds. The expansive cognitive and behavioral effects of gratitude facilitate the creation and preservation of positive social connections, thereby attracting enhanced social support (Fredrickson, 2004; Fredrickson and Branigan, 2005; Wood et al., 2009). In the domain of sports, athletes who possess a strong propensity for gratitude can adopt their coaches’ perspectives during crises within the CAR, acknowledging the coaches’ sincere efforts and dedicating themselves to repairing any rifts (Gan, 2012). This propensity toward gratitude also influences coaches, who respond with proactive prosocial behaviors, nurturing the growth of a harmonious rapport. Additionally, the CAR has been shown to inversely predict burnout, with harmonious interactions enabling athletes to maintain robust relationships and emotional connections with coaches, thus mitigating undue interpersonal stress and curbing the onset of burnout symptoms (Jowett, 2009; Adie and Jowett, 2010; Tabei et al., 2012).

Hope, as elucidated by the broaden-and-build theory, serves as a mediator in the gratitude-burnout relationship. Individuals with a strong disposition toward gratitude are likely to perceive the world more positively and proactively expand their cognitive horizons with an inclusive mindset, thereby effectively managing stress (Fredrickson, 2004; Fredrickson and Branigan, 2005). Research by Chen and Chi (2012, 2015) corroborates that hope and self-confidence are significantly tied to enhanced athletic performance, suggesting that elevated levels of these attributes in college athletes correlate with superior performance outcomes. An augmentation in hope allows individuals to sustain agency and pathways thinking, essential when confronting challenges or stress, safeguarding against negative affectivity and thus forestalling or lessening burnout (Gustafsson et al., 2010). Consequently, psychological interventions targeting burnout could potentially be optimized by concentrating on elevating hope levels, which may improve athletic performance while simultaneously mitigating burnout risks.

### 5.3 The sequential mediating effects of CAR and hope on the relationship between gratitude and burnout

McCullough et al. (2002) contend that gratitude, conceptualized as a moral emotion, serves as a vital cohesive force within social collectives. Within the context of coach-athlete dyads, a harmonious CAR promotes positive and efficacious interactions that alleviate interpersonal tensions, thereby fortifying athletes’ sense of self-identity, clarifying their motivational direction, and amplifying their satisfaction derived from athletic endeavors. This environment is conducive to fostering a hopeful disposition, an optimistic state that buffers against adverse feelings and behaviors (Tennen et al., 2009; Jowett and Nezlek, 2012; Ye et al., 2016a,b). Gustafsson et al. (2010) further reveal that hope robustly negates the propensity for athlete burnout. The present study indicates that athletes endowed with elevated hope are adept at navigating adversities, utilizing optimal strategies to surmount challenges, and maintaining heightened motivation. Hope is instrumental not only in facilitating success when free from impediments but also in proactively addressing and ameliorating psychological distress in response to stressors and adversities, thereby diminishing manifestations of athlete burnout. In summation, both CAR and hope constitute integral components of a ‘mediatory chain’ that links gratitude to athlete burnout, delineating a complex interplay of psychological constructs that underlie the well-being of athletes.

### 5.4 Limitations and future research

This study adopts a cross-sectional design, constraining the extent to which causal relationships can be inferred among the examined variables. Recognizing that athlete burnout fluctuates over time, as suggested by Gustafsson et al. (2010), longitudinal research could offer a more nuanced understanding of its progression. The analysis of the CAR in this study is limited to athletes’ self-reports, omitting coaches’ perspectives, which may provide a more comprehensive overview of the CAR dynamic. Consequently, future studies should consider incorporating matched reports from both coaches and athletes to enrich the understanding of CAR. Methodologically, while the current study constructs a mediation model exploring the interp r mediating roles in the gratitude-burnout nexus, future studies may delve deeper into how gender and sports performance influence these relationships. Our preliminary analyses indicate that these factors might significantly affect the psychological state of athletes. In particular, further research could explore the relationship between technical level and athlete burnout and consider how this relationship may evolve over time.

## 6 Conclusion

The research findings indicate that gratitude, CAR, and hope are integral in attenuating athlete burnout, serving as potent negative predictors. The sequential mediation model elucidated herein demonstrates that gratitude impacts athlete burnout indirectly through CAR and hope, both individually and in combination. These insights provide a theoretical foundation and practical framework for creating interventions aimed at diminishing athlete burnout. To optimize future intervention strategies, enhancing athletes’ gratitude levels, nurturing harmonious CAR, and fostering hope are pivotal. Such measures could not only mitigate the incidence of burnout but also enrich the overall psychological resilience of athletes, thereby contributing to their well-being and performance longevity.

## Data availability statement

The datasets presented in this study can be found in online repositories. The names of the repository/repositories and accession number(s) can be found in the article/supplementary material.

## Ethics statement

The studies involving humans were approved by China University of Geosciences. The studies were conducted in accordance with the local legislation and institutional requirements. Written informed consent for participation in this study was provided by the participants’ legal guardians/next of kin.

## Author contributions

LD: Writing – original draft, Writing – review & editing. RF: Formal analysis, Methodology, Project administration, Supervision, Writing – review & editing. SZ: Supervision, Writing – review & editing. BW: Project administration, Writing – review & editing. LY: Project administration, Writing – review & editing.

## Footnotes

^1^http://www.afhayes.com
